# Early Gene Expression Analysis in 9L Orthotopic Tumor-Bearing Rats Identifies Immune Modulation in Molecular Response to Synchrotron Microbeam Radiation Therapy

**DOI:** 10.1371/journal.pone.0081874

**Published:** 2013-12-31

**Authors:** Audrey Bouchet, Nathalie Sakakini, Michèle El Atifi, Céline Le Clec'h, Elke Brauer, Anaïck Moisan, Pierre Deman, Pascal Rihet, Géraldine Le Duc, Laurent Pelletier

**Affiliations:** 1 Institut National de la Santé et de la Recherche Médicale (INSERM) - Unit 836, Team Nanomedecine and brain, La Tronche, France; 2 European Synchrotron Radiation Facility (ESRF), Biomedical Beamline, Grenoble, France; 3 Grenoble University Hospital, Grenoble, France; 4 Unité Mixte de Recherche 1090, Team Technlogical Advances for Genomics and Clinics (TAGC), Institut National de la Santé et de la Recherche Médicale (INSERM), Marseille, France; 5 Aix-Marseille Université, Marseille, France; 6 Institut National de la Santé et de la Recherche Médicale (INSERM) - Unit 836, Team Functional NeuroImaging and Brain Perfusion, La Tronche, France; 7 Institut National de la Santé et de la Recherche Médicale (INSERM) - Unit 836, Team Synchrotron Radiation and Medical Research, La Tronche, France; Istituto Superiore di Sanità, Italy

## Abstract

Synchrotron Microbeam Radiation Therapy (MRT) relies on the spatial fractionation of the synchrotron photon beam into parallel micro-beams applying several hundred of grays in their paths. Several works have reported the therapeutic interest of the radiotherapy modality at preclinical level, but biological mechanisms responsible for the described efficacy are not fully understood to date. The aim of this study was to identify the early transcriptomic responses of normal brain and glioma tissue in rats after MRT irradiation (400Gy). The transcriptomic analysis of similarly irradiated normal brain and tumor tissues was performed 6 hours after irradiation of 9 L orthotopically tumor-bearing rats. Pangenomic analysis revealed 1012 overexpressed and 497 repressed genes in the irradiated contralateral normal tissue and 344 induced and 210 repressed genes in tumor tissue. These genes were grouped in a total of 135 canonical pathways. More than half were common to both tissues with a predominance for immunity or inflammation (64 and 67% of genes for normal and tumor tissues, respectively). Several pathways involving HMGB1, toll-like receptors, C-type lectins and CD36 may serve as a link between biochemical changes triggered by irradiation and inflammation and immunological challenge. Most immune cell populations were involved: macrophages, dendritic cells, natural killer, T and B lymphocytes. Among them, our results highlighted the involvement of Th17 cell population, recently described in tumor. The immune response was regulated by a large network of mediators comprising growth factors, cytokines, lymphokines. In conclusion, early response to MRT is mainly based on inflammation and immunity which appear therefore as major contributors to MRT efficacy.

## Introduction

Glioblastoma is the most aggressive form of brain tumor. Despite the improvements in therapy [1] the median survival of patients is around 12–15 months after diagnosis with a poor survival rate of 9.8% beyond 5 years.

Synchrotron microbeam radiation therapy (MRT), a new form of radiosurgery, has been applied to rodent brain tumors and composed a new hope of treatment [2], [3]. The use of highly intense synchrotron X-ray beams, with a high energy, high flux and a negligible divergence allows spatial fractionation of an incident beam into arrays of few tens microns wide parallel microbeams, delivering high radiation doses (hGy) in their paths and separated by few hundred microns wide [4].

MRT protocol performed on the brains of adult rats [5], suckling rats [6], ducks embryos [7] and piglets [8] highlighted a sparing effect on normal tissues and can reduce the growth or ablate EMT6 carcinoma [9], SCCVII carcinoma [10] and 9 L intracerebral glioma [6], [11]–[13].

It has been shown that the sparing effect is supported by the radioresistance of normal brain vessels to MRT for doses up to 1,000Gy [14], [15], while there is a denudation of the tumor endothelium and a decrease in tumor blood volume [16], [17]. Beyond the involvement of the vascular component, it has been suggested that other processes were also responsible for tumor control [17], [18] and remain to be described and understood. Besides, the effects of MRT have been studied at *in vivo*, histological or cellular levels, but few information deals with molecular mechanisms. Due to the unique irradiation geometry and the extraordinary dose delivered by MRT, it is not reasonable to extrapolate data and biological molecular events from conventional radiotherapy studies without prior studies.

Describing the early molecular events after MRT could complete the understanding of the normal and tumor tissue response to this particular irradiation. Microarray gene expression technology allows simultaneous analysis of the expression levels of thousands of genes [19]. It has been extensively used to describe the response of biological entity to treatment, to assess genes involved in resistance to therapy and to identify therapeutic targets [20].

The purpose of this work was to characterize the early transcriptomic responses of 9 L tumor and normal brain tissues (contralateral half hemisphere) after a lateral unidirectional MRT exposure (using 50 microns wide microbeams of 400Gy at the tumor location, separated by 200 microns center-to-center). We first illustrated the effectiveness of this single irradiation on the survival of animals. Then, we used oligonucleotide microarray containing 31,100 probe sets (28,000 genes) to acquire the transcriptomic response of both 9 L glioma and contralateral hemisphere 6 h after irradiation. Ingenuity Pathway Analysis software allowed to underline biological functions and canonical pathways involved in MRT response.

## Materials and Methods

### Ethics, animal care and study design

All procedures related to animal care conformed to the Guidelines of the French Government under licenses 380325 and B3818510002 and were approved by the ESRF internal ethics committee named “Internal Evaluation Committee for Animal Welfare and Rights” (IECAWR). The committee specifically approved this study.

All animals were Fischer male rats (Charles River, France) of 8 week-old at arrival. The 9 L cells were implanted one week after the rat arrival (D0), and rats were allocated in two equilibrated groups according tumor size measured using MRI 9 days after implantation (D9). Rats were irradiated 10 days after implantation (D10). The control animals (i.e. non-irradiated) were implanted in common for two experiments including this one, in order to reduce the number of animals.

First, two groups of rats were MRT-treated (n = 20) or not (n = 9) and their survival was measured. Rats were observed and weighted twice a week. They were humanely euthanized (intra-cardiac injection of pentobarbital after isoflurane inhalation) when previously defined clinical criteria were met (prostration, akinesy, epistaxis, rotational motion, 25% body weight loss). In the second part of the study aimed to define the molecular response to MRT, two independent but similar experiments were performed: rats were MRT-treated (n = 5 and n = 5) or not (n = 4 and n = 5) and euthanized after 6 h for brain excision.

Rats were anesthetized with a shot of isoflurane 5% in air prior to an intraperitoneal injection of xylazine/ketamine 64.5/5.4 mg.kg^−1^ for the implantation procedure (and local anesthesia was performed by administration of lidocaine at the top of the scalp) while they were maintained only under isoflurane 2.5% for MRI examination and MRT irradiation. Ocry-gel (Carbopol) was applied to avoid eye deshydration during any anesthesia.

### Tumor implantation

The 9 L cells [20] were grown with complete medium (DMEM/Fetal bovin serum 10%/Penicillin and Streptavidin 1%) at 37°C in a humidified 5% CO2 incubator. As previously described [21], anesthetized Fischer rats were placed on a stereotactic head holder. Then 10^4^ 9 L cells in 1 µL DMEM were injected using a Hamilton syringe into the right caudate nucleus (9 mm anterior to the ear-bars i.e. at *bregma* site, 3.5 mm lateral to the midline, 5.5 mm depth from the skull).

### Tumor MRI examination and rat randomization

Nine days after 9 L implantation, all rats underwent anatomical MRI examination in order to sort them two groups with similar mean tumor size. MRI was performed at 4.7 Tesla or 7 Tesla (Avance III console; Bruker) of the Grenoble IRMaGe MRI facility, using a horizontal magnet and a volume/surface cross coil configuration applying a T_2_ weighted Turbo RARE SE sequence (TE = 33 ms, TR = 4000 ms, field of view = 3×3 cm, matrix: 256×256, slice thickness = 1 mm). The height and width of tumors were measured on the image where the signal modification due to edema and tumor had the largest section. The 3^rd^ direction was estimated by counting the slices displaying the tumor.

### MRT irradiation

Irradiations were performed at the ID17 Biomedical Beamline of the European Synchrotron Radiation Facility (ESRF, France) using X-rays emitted tangentially from electron bunches circulating in a storage ring. The wiggler produces a wide spectrum of photons which extends, after filtration, from 50 over 350 keV (median energy: 90 keV) [22]. The mean dose rate was then ∼62Gy.mA^−1^.s^−1^ allowing very fast irradiation. The quasi-laminar beam was micro-fractionated into an array of 41 rectangular and quasi-parallel 50 microns width microbeams, separated by 200 microns center to center each. This was performed by using the ESRF Multislit Collimator positioned 33 m from the photon source and 80 cm upstream from the rat holder [23]. Ten days after tumor inoculation, the animals were positioned prone on a Kappa-type goniometer (Huber, Germany) in front of the x-rays source, on a home-made plexiglas frame and the alignment into the beam was performed using live cameras. The contention of the rats was performed by a teeth bar while the animals were under anesthesia. They were placed perpendicularly to the beam and received a lateral irradiation, from their anatomical right to left side. The beam was shaped into a field of irradiation of 8 mm horizontal and the animals were scanned vertically over 10 mm through the beam after opening of the shutter. Although the total procedure lasted about 2 min for each rat, the irradiation time is around to 2 s. Animal immobility during exposure was checked on three control video screens located in the control hutch.

### Dosimetry and ballistic of irradiation

The microbeam dose at the tumor (*i.e.* 7 mm of depth from lateral side) was 400Gy, the valley dose was 18±0.6Gy as computed by Monte Carlo simulations [24]. The spatial configuration of irradiation was checked by radiochromic films (Gafchromic, HD-810) exposed in front of rats. The conservation of spatial configuration in tumor depth was checked by pH2AX staining (double strand break indirect staining) on tissue sections 6 h after irradiation.

### Survival curves

Kaplan Meier survival data of 9 untreated rats and 20 treated rats was plotted versus time after tumor implantation. Median Survival Time (MST) and statistical analysis (log rank test) were performed using GraphPad Software, USA.

### Brain excision

Untreated and MRT-treated rats (respectively n = 10 and n = 9) were sacrificed 6 h after irradiation, and the brain of each animal was immediately frozen in liquid isopentane at −50°C and stored at −80°C.

### Total RNA extraction and quality control

For each brain, 25 coronal sections slices of 60 µm thick were cut at −20°C on a cryostat (Microm HM80, France). The tumors and the contralateral tissues were isolated using a micropunch and kept in lysis RNAse free buffer from the MirVana isolation kit™ (Ambion, Applied Biosystems, Foster City, CA).

Total RNA of each tissue was extracted with the previous kit according to specifications. RNA integrity and concentration were checked by Agilent NanoRNA Chip (Bio-Analyser, Agilent Technologies, Palo Alto, CA, USA). A minimum RNA Integrity Number of 7.6 was required for all samples.

### RNA microarray

**Figure 1:**
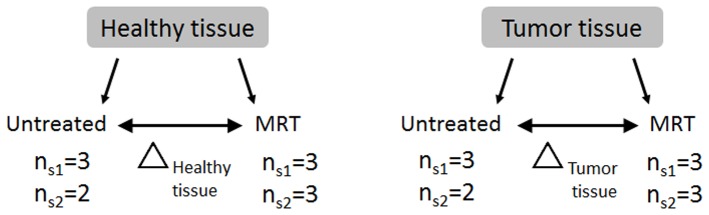
Experimental scheme. Contralateral normal and tumor tissue samples were irradiated (MRT) or not (untreated) in 2 experimental sessions (S_1_ and S_2_). The transcriptomic data were compared in order to determine contralateral normal and tumor tissue responses to MRT (Δ _normal tissue_ or Δ _tumor tissue_).

Microarray analysis were performed on total RNA of brain of untreated (n = 5) and MRT-treated groups (n = 6) from two experimental sessions (Figure 1). For each sample, 200 ng of total RNA was amplified with the GeneChip 3′IVT Express Kit (Affymetrix, Santa Clara, CA) and hybridized on GeneChip® Rat Genome 230 2.0 Array according to Affymetrix specifications. Briefly, mRNAs were reverse transcribed to double stranded cDNA, amplified, fragmented and biotin-labelled. End-label cDNA were hybridized to microarray chip for 16 h at 45°C and 60rpm. After washing and staining in Affymetrix GeneChip® Fluidics Station 450, microarrays were scanned using Affymetrix GeneChip® Scanner 3000. Light emission at 570 nm is proportional to each oligonucleotide amount on the GeneChip® array.

### Gene expression normalization

Background adjustment and normalization of all raw probe intensity were performed using the Robust Multi-array Average (RMA) algorithm [25] implemented in Affymetrix Expression Console. The expression values were reported in arbitrary units. Moreover, the MicroArray Suite 5 (MAS5) algorithm [26] was used on raw data to identify probe sets which were out of the limit of detection for the system and to flag the transcript as Present (P) or Absent (A). P- and A-flagged information was used to filter data in order to remove false positive after statistical analysis.

### Statistical analysis of microarray data

Statistical analysis was performed using the TIGR MultiExperiment Viewer version 4.5.1 software (TMeV, http://www.tm4.org/mev/). Significant differentially expressed genes between the two tumor groups and between the two contralateral tissue groups (MRT treated or not) obtained 6 h after irradiation were assessed using an unpaired Significance Analysis of Microarrays (SAM) [27]. A False-Discovery Rate (FDR) lower than 5% was fixed to generate significant genes list. Hierarchical clustering was directly generated thanks to TMeV from this list. For all other analyses, probes were only considered when at least n-1 values were P-flagged (MAS5) in any of both compared groups.

### Functional analysis

The molecular & cellular functions and canonical pathways associated with differentially expressed genes identified by SAM test (FDR 5%) and P/A flag filtered were identified thanks to Ingenuity Pathways Analysis (IPA) software (Ingenuity® Systems, www.ingenuity.com). The data set was restricted to mammal species.

Molecules from the data set were associated with the most relevant “molecular & cellular functions” and “canonical pathways” in the Ingenuity Knowledge Base. The significance of the association between the data set and the bio-function or the canonical pathways was measured in 2 ways: 1) a ratio between the number of molecules for a given function or pathway issued from the data set and the total number of molecules for the same function or pathway is displayed. 2) Right-tailed Fisher's exact test was used to calculate a p-value determining the probability that each function or pathway assigned to that data set was due to the chance alone. For canonical pathways, a false discovery rate of 1% was further applied to correct for multiple testing [28], [29].

### RT-qPCR transcriptional validation for genes regulated 6 h after MRT

Some selected significantly up- and down-regulated genes were validated using reverse transcription and quantitative polymerase chain reaction (RT-qPCR) on untreated and MRT-treated tumor samples (7<n<10, depending on sample availability).

Two micrograms of total RNA were transcribed into cDNA using Promega Reverse Transcription reagents with random dN6 primers. Specific gene primers (Table 1) were designed using software (https://www.roche-applied-science.com/sis/rtpcr/upl/ezhome.html). Real-time PCR were performed according to the SYBR Green method on an Mx3000™ apparatus (Stratagene, La Jolla, CA) using Quantitect SYB® reagents (Qiagen, France). Data were normalized using two housekeeping genes, Atp5b and Arpc1a, selected according to Affymetrix data because of both their suitable range of quantification and very low variation in expression levels across all samples (8% and 7%, respectively). Thermal-cycling parameters were as follows: denaturation at 95°C for 10 min, cycling regime of 40 cycles at 95°C for 15 s, 56°C for 30 s, and 72°C for 30 s.

**Table 1:** Validation gene set.

Symbol	Gene Name	Forward (5′-3′)	Reverse Primer (5′-3′)
**Arpc1a**	actin related protein 2/3 complex, subunit 1A	gtttgctgtggggagtgg	ggatcggcttcttaatgtgc
**Atp5b**	ATP synthase, H+ transporting, mitochondrial F1 complex, beta polypeptide	gggtacaatgcagga	tcagctggcacatag
**Fam64a**	family with sequence similarity 64, member A	gaagctgtctcaaaagctgga	aagggagacggtcatgtcac
**Inhbe**	inhibin beta E	caggcagcactgaccaga	gcggtaggttgaagtggatt
**Mars**	mehionine-tRNA synthetase	atacgttcggtcgcacaac	gcaacctctggaagatgtcc
**Traf4af1**	TRAF4 associated factor 1	cggaggaacatcagaagcag	gctcgtttttatccttcagatcc
**Pttg1**	pituitary tumor-transforming 1	ttcttccccttcgatcctct	aggggagaagtgagatctggt
**Trib3**	tribbles homolog 3b	tcaagttgcgtcgatttgtc	ccagtcatcacacaggcatc

List of the genes with significant up- or down-regulation which were selected for microarray data validation using RT-qPCR. Primer sequences for quantitative RT-qPCR are indicated in the two right-hand columns.

## Results

### Survival

**Figure 2:**
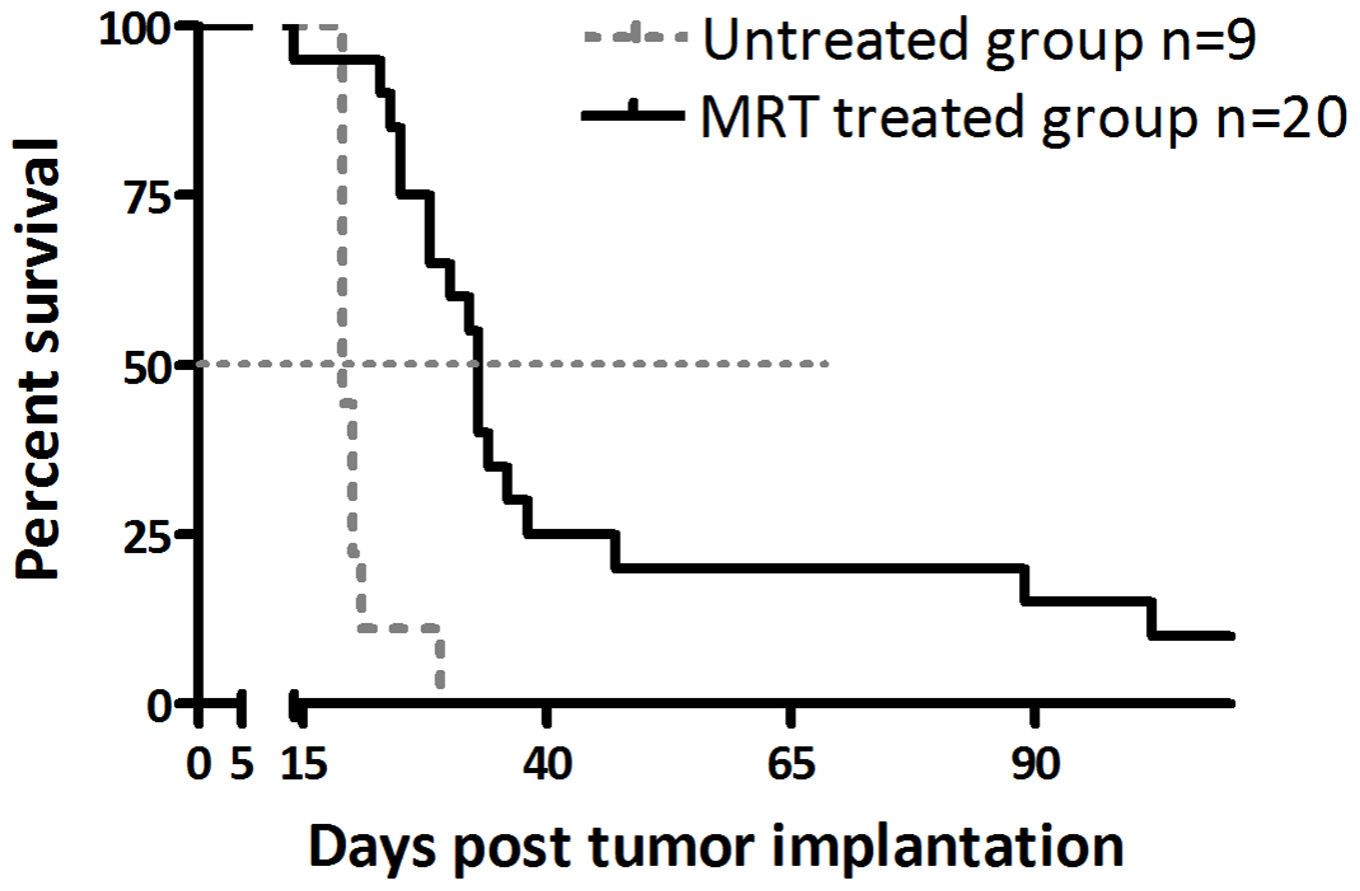
Kaplan-Meier representation of tumor-bearing rat survival. Intracerebral 9-irradiated (solid black line) or not (dashed grey line). MRT significantly increased the Median Survival Time of animals compared with untreated rats (33 days *versus* 19 days, log rank test: p<0.0001).

In order to obtain similar irradiation in tumor and normal contralateral brain tissue, we have adapted a configuration of MRT previously reported to have a high interest for 9 L brain tumors therapy [17]. As shown in figure 2, this unilateral MRT irradiation significantly increased the Mean Survival Time (MST) of treated animals compared with the untreated group (33 versus 19 days respectively, p<0.0001).

### Identification of early gene response in 9 L tumor and contralateral brain tissue

**Figure 3:**
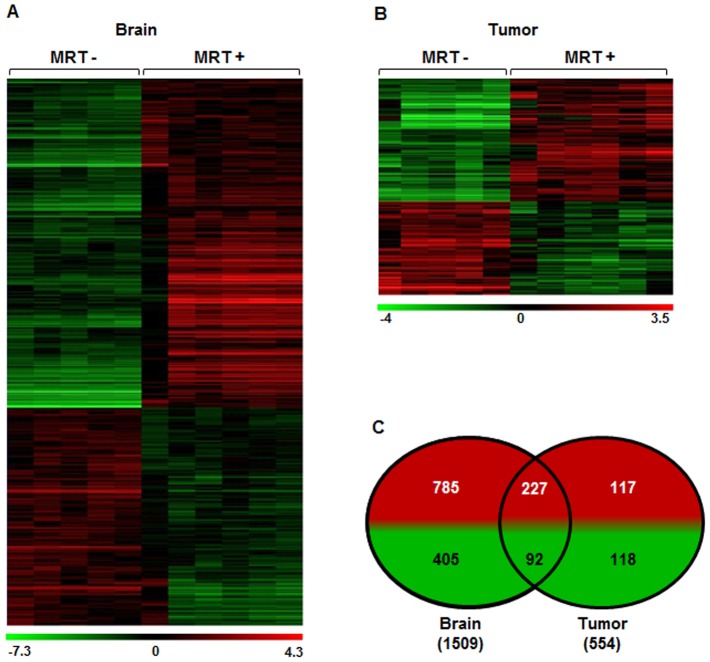
Influence of MRT on gene expression in tumor and contralateral brain tissues. a, b- Heat map showing either significant decrease or increase in mRNA expression after MRT in tumor and normal brain tissues. Colors indicate expression levels above (red) or below (green) the median value for each gene. Vertical columns indicate individual arrays and horizontal rows indicate genes. c- Venn diagrams showing the numbers of significantly increased (red) or decreased (green) genes after MRT in both tissues.

Six hours after irradiation, we observed (i) a modification of transcriptomic profiles in both tissues and (ii) a difference in responses of tumor and contralateral tissues (Figure 3a and b). Indeed, 1,509 genes (1,012 were induced and 497 were inhibited) and 554 genes (344 were induced and 210 were inhibited) significantly responded to MRT in tumor and contralateral tissues, respectively (Figure 3c and Table S1). Figures 3a and 3b show the clustering analysis based on the selected genes in contralateral and tumor tissues respectively. Among them, 319 were common to both tissues as represented on Venn diagram (227 were induced and 92 were inhibited).

### Validation of gene expression modulation by RT-qPCR

**Figure 4:**
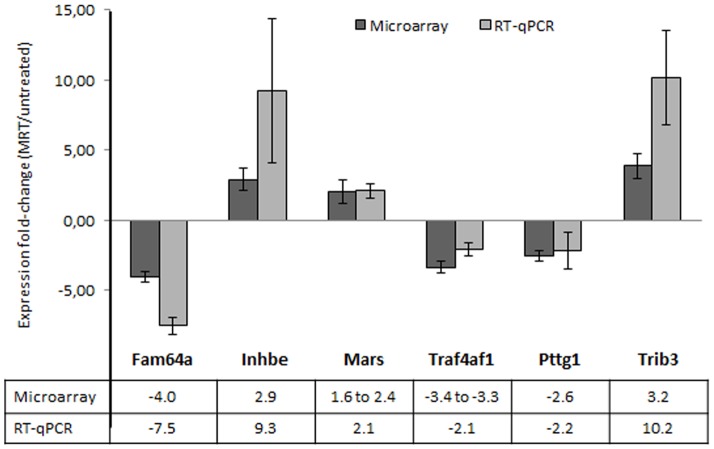
Validation of microarray analysis by quantitative RT-PCR on tumor tissue samples. Data were first normalized to the expression of Arpc1 and Atp5b housekeeping genes. The fold changes (±SEM) in each gene expression was calculated using the mean expression in treated (n = 6 for microarray and n = 8 to 9 for RT-qPCR) versus untreated (n = 5 for microarray and n = 7 to 10 for RT-qPCR) tumors 6 h after MRT. Fold changes are indicated below the histograms (several fold changes were available for microarray because of the presence of several probesets). All tested genes presented a significant difference between treated and untreated tissues (p<0.05; permutation t-test).

We validated the variation of gene expression by quantitative RT-PCR on 6 representative genes with significant expression modulation in tumor. RT-qPCR analysis was performed on tissues that had been already analyzed on microarray and extended on supplementary tumors (in all n≥7). The RT-qPCR results confirmed that expression of all selected genes was significantly different between treated and untreated tumors (p<0.05, permutation t-test) and were therefore consistent with those obtained by microarrays (Figure 4).

### Identification of biological functions modulated in 9 L tumor and contralateral brain tissues 6 hours after MRT

**Figure 5:**
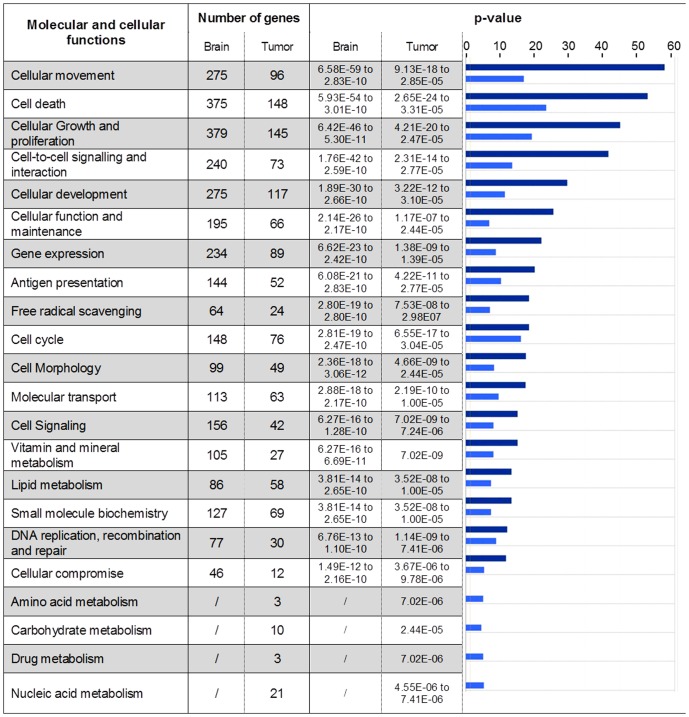
Molecular and cellular functions constituting the MRT response. Genes with a significantly modified expression in brain or tumor 6-values (−log(p-value)) are illustrated for each function. The p-values associated with brain (dark blue) and tumor (light blue) are represented using histogram in the right-hand column. The p-values were calculated using the right-tailed Fisher's exact test in IPA.

Genes identified by SAM analysis with a 5% FDR were classified into different bio-function categories based on IPA database (Figure 5). The response to MRT irradiation involved 22 and 18 molecular and cellular functions in tumor and normal brain tissues, respectively. All functions found in contralateral tissue were also represented in tumor tissue. Some bio-function categories such as cell death, growth, proliferation, cell cycle, cellular function and maintenance can contribute to cell response to radiation-induced damages. Inflammation and immunity reaction appeared as a common point of cellular movement, antigen presentation and cell-to-cell signaling/interaction categories. Despite a lower number of genes per group, tumor response to MRT displayed four supplemental bio-functions compared to normal tissue: amino acid metabolism (p = 7.02e^−06^), carbohydrate metabolism (p = 2.44e^−05^), drug metabolism (p = 7.02e^−06^) and nucleic acid metabolism (p = 4.55e^−06^). These 4 bio-functions showed the lowest p-value obtained for MRT response in tumor (Figure 5).

### Identification of pathways modulated in 9 L tumor and contralateral brain tissues 6 hours after MRT

Genes modulated 6 hours after MRT in tumor and contralateral tissues were grouped in canonical pathways (Table S2). Three statistical stringency levels were applied to data with 5, 1 and 0.1% of False Discovery Rate (FDR), corresponding to p-values lower to 1.62E-2, 2.57E-3 and 1.45E-4 respectively. The number of pathways ranged from 83 (FDR0.1%) to 165 (FDR5%) in contralateral brain, from 36 to 100 in tumor and from 86 to 170 when both tissues were considered together. Pathways were more numerous for brain than for tumor (>1.6), as already observed for modulated genes. For all FDRs, more than half of all pathways were found in both tissues (55.3% for FDR5%, 50.4% for FDR1% and 73.3% for FDR0.1%). So the response to MRT appeared to mainly involve similar mechanisms in normal brain and tumor. More, whatever the tissue or the FDR, pathways related to immunity or inflammation represented more than 55% of the total (from 55.8 for brain and tumor tissue with FDR5% to 74.4% for brain and tumor tissue with FDR0.1%). Thus the response to MRT was mainly based on these two processes. The pathways related to immunological or inflammatory responses were enriched in the most stringent statistical conditions, illustrating that these pathways are among the most significant ones.

We focused analysis on pathways with FDR1%. Genes modulated by MRT were grouped on 128 pathways in normal brain, 73 in tumor and 67 were common to both tissues. Among them, 82 and 49 pathways were related to immunity or inflammation in normal brain and tumor respectively. Forty six immune-relative pathways were common to both tissues; however they could include different genes for tumor and contralateral tissues (Figure 5).

All domains of innate and adaptive immunity were found in common canonical functions: macrophages (“Role of Macrophages, Fibroblasts and Endothelial Cells in Rheumatoid Arthritis”, “Production of Nitric Oxide and Reactive Oxygen Species in Macrophages”, “IL-12 Signaling and Production in Macrophages”), natural killer cells (NK, “Crosstalk between Dendritic Cells and Natural Killer Cells”), dendritic cells (“Dendritic Cell Maturation”, “Crosstalk between Dendritic Cells and Natural Killer Cells”), T lymphocytes (“Altered T Cell and B Cell Signaling in Rheumatoid Arthritis”, “iCOS-iCOSL Signaling in T Helper Cells”, “4-1BB Signaling in T Lymphocytes”) and B lymphocytes (“Altered T Cell and B Cell Signaling in Rheumatoid Arthritis” and “B Cell Receptor Signaling”, “PI3K Signaling in B Lymphocytes”, “B Cell Activating Factor Signaling”). Many canonical pathways and especially those centered on cytokines signaling, evoked also the immune network: Interleukins 1, 6, 8, 9, 10, 12, 15, 17A and 17F were found in 13 pathways. Immune cell communication was illustrated by other either mediators or receptors: TNF (“TWEAK Signaling”, “April Mediated Signaling”, “4-1BB signaling in T Lymphocytes”), Interferon (“Role of PKR in Interferon Induction and Antiviral Response”, “Role of JAK1, JAK2 and TYK2 in Interferon signaling”) and others (“iCOS-iCOSL Signaling in T Helper Cells”, “MIF Regulation of Innate Immunity”, “CD40 Signaling”). As expected, inflammation was found (“Acute Phase Response Signaling”, “TREM1 Signaling”, “Atherosclerosis Signaling”, “Role of IL-17F in Allergic Inflammatory Airway Diseases”, “Pathogenesis of Multiple Sclerosis”, “Role of IL-17A in Arthritis”, “MIF-mediated Glucocorticoid Regulation”, “Glucocorticoid Receptor Signaling”).

The common response to MRT comprised also modifications in the expression of genes involved in other mechanisms: Cell cycle, cell growth, apoptosis (“Induction of Apoptosis by HIV1”, “Apoptosis Signaling”, “Death Receptor Signaling”, “p38 MAPK Signaling”, “p53 Signaling”, “PI3K/AKT Signaling”), DNA damage sensing and repairing (“ATM Signaling”) and vascular physiology or angiogenesis (“Erythropoietin Signaling”, “Renin-Angiotensin Signaling”, “Angiopoietin Signaling”).

In normal brain, other pathways for cell cycle/growth or apoptosis (“Cell Cycle Regulation by BTG Family Proteins”, “ERK/MAPK Signaling”, “PTEN Signaling”), DNA damage sensing or repairing (“Role of BRCA1 in DNA Damage Response”) and vascular physiology and angiogenesis (“Role of Tissue Factor in Cancer”) were found.

Of interest, in tumor tissue, we found one pathway associated with both DNA damage and cell cycle regulation (“Cell Cycle: G2/M DNA Damage Checkpoint Regulation”).

A part of pathways were found in only one tissue. Concerning normal brain, a high proportion of pathways related to T-cells (15/49) was observed in comparison with those found in both tissues (6/46). On the opposite, only 4 pathways were preferentially associated with innate immunity in normal brain tissue.

## Discussion

In this work we characterized the transcriptomic responses of tumor and normal brain tissues 6 hours after an MRT irradiation. A suitable model should heed following parameters: both tissues should originate from the same species in order to use a unique microarray type for transcriptome analysis, and host animal should be immunocompetent for considering the influence of the immune system. The 9 L tumors orthotopically implanted in syngeneic Fisher rat brain fulfill these conditions [20], [30].

MRT has already been demonstrated as a promising irradiation modality for brain tumors therapy at preclinical level. Although MRT crossfire irradiation, the most efficient configuration for improving survival to date [6], implies that the tumor and the contralateral brain tissues are not treated the same way. We chose therefore a simpler configuration with a lateral unidirectional MRT irradiation in order to apply similar irradiation configuration in both tissues. This 400Gy in-microbeam dose scheme, applied to rat brain 10 days after tumor inoculation, significantly increased (×1.7) the median survival time (MST) compared to untreated rats (Figure 2). The set parameters enable then a rigorous analysis of biological mechanisms of MRT impact in both tumor and normal brain tissue responses. Although several studies already aimed at the understanding of biological processes induced by MRT, they were essentially based on late events (from 48 h post-MRT) [16], [17]. In this work we focused on early response, assuming that early transcriptomic events would dictate later modifications at molecular, cellular, tissular and finally therapeutic levels. We considered also the particular lack of knowledge concerning MRT-associated biology at transcriptomic level.

MRT differs from conventional irradiation because it delivers several hundreds of grays in micrometric volumes in few seconds with a particular geometry, which might influence the gene expression response. Due to recent improvements in theoretical and experimental dosimetry [31] experiments involving broad beam irradiation with synchrotron sources are currently in progress in our laboratory to determine the impact of the complex irradiation scheme of MRT. Although a similar study has been done in Boomerang (Melbourne, Australia) [32], the experimental parameters such as the source, the MRT parameters, the tumor model differ than ours.

We determined the pan-genomic response of both tumor and normal brain tissues 6 h after MRT by using microarrays containing 31,000 probesets. In normal brain tissue the expression of about 5% of genes was modified. This proportion is higher than those described using other methods after ionizing irradiation of brain (0.6% 8 h after 10Gy [33], 1.1% and 2.2% 5 h after 10Gy and 20Gy [34]) but is consistent with a previously reported dose-dependent increase in both expression level and number of modulated genes [34]–[36]. So the unusual number of engaged genes after MRT could be linked to the high dose deposited in tissue, from ∼18Gy in valleys to 400Gy in microbeams.

In tumor 1.8% of genes were modified. To our knowledge, no microarray analysis was performed in 9 L after irradiation and those conducted on other high grade glioma models were very different to ours in number of analysed genes, type and dose of irradiation. The unique comparable study applied to MRT was performed on EMT6 subcutaneous mammary tumor [32]. It revealed the modulation of 184 genes but did not address the response of normal tissue.

The response to MRT of normal brain tissues involved 2.7 times more genes than the one of tumor tissue (1,509 versus 554), revealing a tissue-specific response to MRT (Figure 3 and Table S1). Such a higher number of genes could arise from the higher cellular diversity of normal tissue, due to the presence of specific parenchymal and stromal cells such as neurons, astrocytes and oligodendrocytes. Surprisingly these genes were associated with less molecular and cellular functions in normal (18 functions) compared with tumor tissue (22 functions; Figure 5). The 4 supplementary functions in tumor were associated with metabolic processes. However they displayed the lowest statistical significance for tumor response. Responses of both tissues appeared therefore to be close to each other since 18 functions were common. Moreover, a modulation of genes involved in mitosis, cell cycle regulation, apoptosis, cell adhesion, glycolysis, lipid metabolism, has already been reported after ionizing radiation [37] and could be connected to many functions implicated in the response of the both tissue after MRT.

A large number of canonical pathways were also identified 6 h after MRT: 128 and 73 in tumor and contralateral tissues, respectively (Table S2). Among them, 67 were common. More than 90% of canonical pathways found in tumor were also found in brain (only 6 specific pathways out of 73), while 52.3% of normal brain pathways were found in tumor. Thus the tumor response to MRT was mostly included in the brain tissue one. Again, this result could reflect a higher heterogeneity in cell composition and functional processes in normal brain compared to tumor.

Of interest, we observed only one canonical pathway related to brain parenchyma response to MRT (“Huntington's disease signaling”) which includes genes related to apoptosis (Caspase 1, 3, 7, 8, 12, bax, TP53), intracellular signaling (GNG2, GNG11, CREB3, PRKCH, PRKCD) and one is neuron-specific (CACNA1B). This canonical pathway suggests that some neurons undergo apoptosis in response to MRT.

Among the 67 common pathways to both tissues, immunity and inflammation were widely represented. Modulation of immunity and inflammation has been often reported in brain in response to various stimuli or injuries: Presence of a tumor mass, radiotherapy [38], bacterial or viral infection [39], neurodegenerative disorders such as Alzheimer's and Parkinson's diseases ([40], [41]).

But our results are also in agreement with previous works which reported the immune activation in response to conventional irradiation and even considered irradiation as an “immunological adjuvant” [38], [42]–[44]. Concerning MRT, previous works mainly focused on vascular parameters to explain therapeutic effect [17]. However increasing leukocytes recruitment was observed in both normal brain [45] and 9 L tumor tissues [17]. Moreover, Sprung *et al.* recently reported that modifications in immunity-related gene expression is a hallmark of response to MRT in mouse mammal tumor [32].

Gasser *et al.* [46] reported that DNA damage can elicit a response of immune system, and in particular of innate compartment (macrophages). But other normal cells constituents can be released into the extracellular compartment during states of cellular stress or damage and subsequently activate inflammation and immunity. Among these Damage-Associated Molecular Patterns, we found HMG1 that is both involved in infectious and sterile inflammation, immune response and tissue repair or regeneration [47]. Receptors such as Toll-like receptors, C-type lectins are sensing these damage-associated mediators. Indeed, we observed a modulation in the expression of several potential mediators and receptors involved in the simulation of inflammation or immunity. For example, HMGB1, Toll-like receptors 1, 2, 7, C-type lectins 7A and CD36 and other altered constituents of irradiated tissue can trigger activation of innate or adaptive cells. These genes may serve as a link between biochemical changes in response to irradiation and the inflammation or immunological challenge.

We evidenced subsequently to this activation that most populations of the immunological compartment were triggered (macrophages, natural killer, dendritic cells, T and B lymphocytes) and many direct crosstalks between cells or diffusible mediators highlighted communication between them.

Dendritic cells constitute the first line of the adaptive immune response, as antigen-presenting cells. Maturation of dendritic cells has been reported to be impaired in cancer in response to tumor-derived mediators, especially the Vascular Endothelial Growth Factor [48]. However the modulation of the pathway “maturation of dendritic cells” in our study suggests that dendritic cell maturation occurred in response to MRT, likely as a part of the “immunological adjuvant” phenomenon discussed above. This would participate in enhancing efficiency of immune control of cancer progression.

We observed also at transcriptomic level the recruitment of innate immune compartment. Presence of Natural killer (NK) was indicated in our study by the modulation of two canonical pathways (“Crosstalk between Dendritic Cells and Natural Killer Cells”, “Tumoricidal Function of Hepatic Natural Killer Cells”) and could be a crucial point for MRT efficiency since NK were previously shown to eliminate 9 L glioma cells both *in vitro* [49] and *in vivo* [50], [51].

Among the large spectrum of diffusible mediators, one of them, IL-17 is specifically expressed by T helper 17 cells (Th17; [51]). The presence of these cells was reinforced by modifications in expression of IL-6, IL-23, STAT3, ICOS. These cells are key mediators of a broad array of inflammatory or autoimmune diseases and have been extensively found in tumor microenvironment [52]. But their positive or negative role in tumor progression is still debated. The recruitment of Th17 cells was reported to be triggered by local inflammation at the tumor site [53]. One can hypothesize that irradiation could increase inflammation and therefore Th17 cell recruitment.

Our transcriptomic results also indicate the presence of cytotoxic T cells (“Cytotoxic T Lymphocyte-mediated Apoptosis of Target Cells” (Table S2). In parallel with NK cells, cytotoxic T cells were heavily reported in immunological antitumor phenomenon. Cytotoxic T cells have been associated with spontaneous tumoricidal action on glioma and emerged as a part of some therapeutic strategies. For example, vaccination of rats with dendritic cells coinjected with processed GM-CSF secreting 9 L cells triggered the regression of distant 9 L tumors [54]. Treatment efficacy was associated with a Th1 response and thus IFNγ secretion [55]. IFNγ appears also in several canonical pathways modified by MRT in our study (Table S2).

In conclusion to this work we report an extended gene expression profile associated with the MRT responses in both normal and tumor tissues. The early transcriptomic response is very similar in both tissues, mainly involves modifications associated with immunity and inflammation. The detailed study of pathways modulated by MRT reveals the involvement of transcriptomic modification in relation with innate and/or the adaptative immune response. More specifically, pathways and biofunction in link with NK or CD8+ T lymphocytes are particularly reprensented. Further immunological studies and functional analysisare needed for evaluating the role of those immune mechanisms in the therapeutic impact of irradiation. This is an important step for understanding the biological mechanisms responsible for the therapeutic index of the MRT.

## Supporting Information

Table S1 **Genes responding significantly to MRT in tumor and normal brain tissues.** List of all genes which expression is significantly modified in brain (1,509 genes) and tumor (554 genes). Probesets are ranked for each tissue in descending order of expression MRT/untreated ratio.
(XLSX)

Table S2 **Canonical pathways constituting the MRT response.** All canonical pathways (IPA) found statistically significant with 1% FDR (corresponding to p-value lower than 2.57E-3) are listed for both normal and tumor tissues.
(XLSX)
